# Increased collateral flow by rescue femorofemoral bypass dissolved residual thrombus in implanted iliofemoral artery stents: a case report

**DOI:** 10.1186/s13256-021-02794-6

**Published:** 2021-04-27

**Authors:** Tatsuo Haraki, Taichi Kondo, Izaya Kamei, Takahiro Tanabe

**Affiliations:** Department of Cardiology, Saitama Eastern Cardiovascular Hospital, 3187-1, Osawa, Koshigaya-City, Saitama 343-0025 Japan

**Keywords:** Femorofemoral bypass, Deep femoral artery, Thrombus, Acute limb ischemia, Stents, Collateral flow

## Abstract

**Background:**

Failed aortofemoral and femoropopliteal bypass grafts in the lower extremity artery usually result in acute limb ischemia. Endovascular treatment and surgical revascularization have been reported for limb salvage.

**Case presentation:**

A 72-year-old Japanese man was admitted with acute limb ischemia due to failed aortofemoral and femoropopliteal bypass grafts. Endovascular treatment with balloon angioplasty, thrombectomy, and stent implantation in the long chronic total occlusion from the right common iliac artery to the superficial femoral artery did not result in efficient flow due to thrombus transfer from a failed aortofemoral bypass graft. However, a rescue femorofemoral bypass (the left femoral to the right deep femoral artery) improved his symptoms, and implanted in-stent flow was gradually recovered. Lower extremity angiography performed 5 months later confirmed the patency of the iliofemoral in-stent flow. However, the femorofemoral bypass graft was unfortunately occluded due to the progression of left external iliac artery stenosis. The patency of the iliofemoral in-stent flow was confirmed at 1 year by ultrasonography.

**Conclusions:**

Improvement of the deep femoral artery flow plays an important role in the treatment of acute limb ischemia due to failed aortofemoral and femoropopliteal bypass grafts. Thus, increased collateral circulation to the periphery through the deep femoral artery dissolved the remaining in-stent thrombus in the iliofemoral artery.

## Background

Acute limb ischemia (ALI) requires prompt, appropriate treatment because it occurs rapidly, is life-threatening, and can lead to the amputation of the lower extremities [1]. Aortofemoral and femoropopliteal bypass graft failure usually results in ALI. Endovascular treatment (EVT) including thrombectomy, surgical revascularization using a Fogarty catheter, femorofemoral reoperation, and femoropopliteal and axillary-femoral bypass, has been reported for ALI treatment for failed grafts [2–4]. Catheter-directed thrombolysis (CDT) is also a key therapeutic approach, but it can have significant clinical complications, including severe systemic or intracranial bleeding [5, 6].

We experienced a case of ALI due to failed aortofemoral and femoropopliteal bypass grafts. EVT in the long chronic total occlusion (CTO) from the common iliac artery (CIA) to the superficial femoral artery (SFA) did not result in efficient flow despite the implantation of stents in the CIA, external iliac artery (EIA), and SFA, particularly due to unexpected thrombus movement from the aortofemoral graft during balloon angioplasty. However, a rescue femorofemoral bypass gradually dissolved the thrombus in the implanted iliofemoral stents.

## Case presentation

**Fig. 1:**
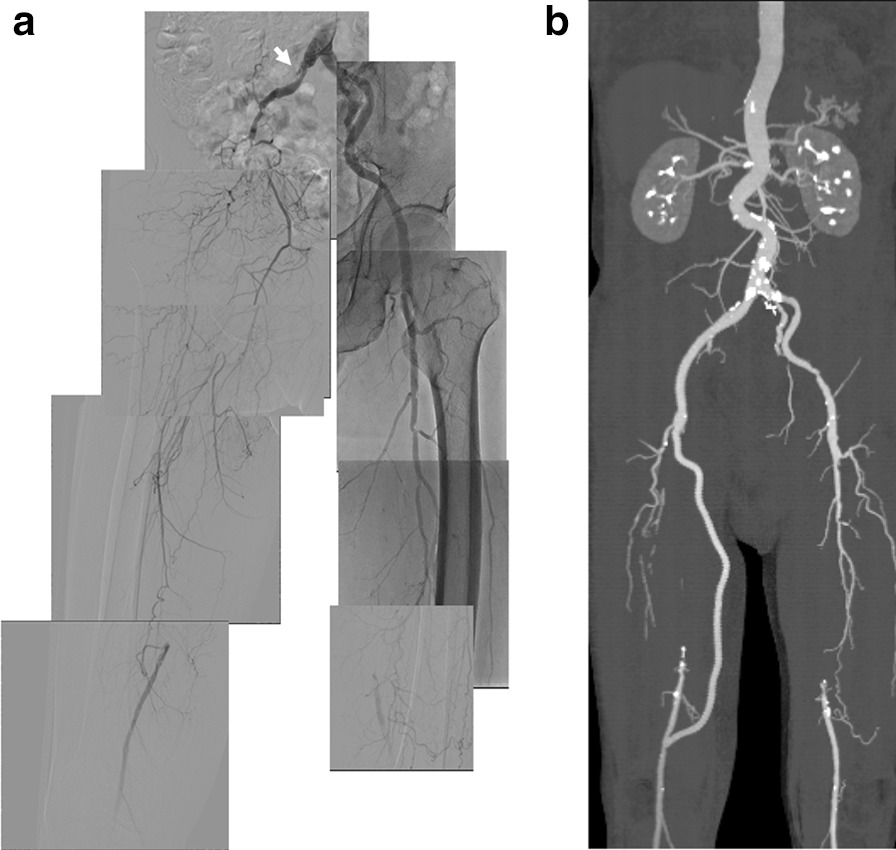
Lower extremity angiography and multislice computed tomography (MSCT) at the first hospitalization. **a** Lower extremity angiography in January 2019. The right external iliac artery was occluded (white arrow), and the right superficial and deep femoral arteries were also occluded. The right femoral artery was mainly maintained by collateral flow from the right internal iliac artery. **b** After aortofemoral and femoropopliteal bypass grafts

A 72-year-old Japanese man with a medical history of hypertension had felt intermittent claudication of the right leg beginning in autumn 2017, and visited our hospital when his symptoms worsened in January 2019. He had a history of hypertension, and had taken amlodipine (5 mg per day) and valsartan (40 mg per day) for 20 years. He had bladder carcinoma in 2018. His father also had a history of hypertension. He smoked one pack of cigarettes per day for about 50 years, and he was a social drinker. He was an office worker. His right ankle-brachial index (ABI) was 0.33. Multislice computed tomography (MSCT) and angiography of the lower extremity showed occlusion from the right EIA to the SFA, and occlusion of the left SFA (Fig. 1a). His right femoral artery was mainly maintained by collateral flow from the right internal iliac artery (IIA). An aorto-right femoral and right femoropopliteal bypass using polytetrafluoroethylene (PTFE; Gore Propaten 8 and 6 mm) was then performed in February 2019 (Fig. 1b). He received dual-antiplatelet therapy of aspirin (100 mg per day) + cilostazol (100 mg per day) and telmisartan (40 mg per day) after surgery.

The patient suddenly developed a cold sensation in the right lower limb, foot pain, and intermittent claudication 1 year later. An MSCT in another hospital 5 days after onset showed total occlusion in both the aortofemoral and femoropopliteal grafts. The peripheral blood flow of the distal SFA and below-the-knee vessels were mainly nourished by collateral circulation from the lumbar artery. Thus, the patient was again referred to our hospital. On admission, the patient’s vital signs were stable, and his blood pressure in the right arm was 145/68 mmHg, pulse rate was 77 beats/minute, oxygen saturation was 98% on room air, and body temperature was 36.3 °C. The skin color of the right lower limb was pale, and the skin temperature was significantly reduced, particularly in the lower leg. There was no motor or sensory loss. His dorsalis pedis artery and the posterior tibial artery of the right lower limb were not palpable or audible by Doppler. His right ABI was not calculated.

A chest X-ray demonstrated no abnormal findings. Electrocardiography showed normal sinus rhythm and no ST-T segment changes. The laboratory examination findings showed that most parameters were within normal limits: white blood cell count 6.4 × 10^3^/μL, red blood cell 5.07 × 10^6^/μL, platelets 19.3 × 10^4^/μL, C-reactive protein 0.30 mg/dL, serum creatinine 0.92 mg/dL, creatine phosphokinase (CPK) 115 U/L, aspartate aminotransferase (AST) 19 U/L, alanine aminotransferase (ALT) 15 U/L, lactate dehydrogenase (LDH) 211 U/L, LDL cholesterol 110 mg/dL, HDL cholesterol 60 mg/dL, plasma glucose 115 mg/dL, HbA_1c_ 6.1%, prothrombin time/international normalized ratio (PT-INR) 1.02, and activated partial thromboplastin time (APTT) 29.8 seconds, except for the increased levels of D-dimer 2.9 μg/ml and fibrin degradation products (FDP) 14.7 μg/ml.

An infusion of heparin and prostaglandin E1 (PGE1) was started, but his symptoms did not resolve. Reoperation (axillary-femoral or femorofemoral bypass) or CDT in the bypass graft was considered. However, the patient did not wish to have surgery again, because he had intestinal hernia complications after aortofemoral and femoropopliteal bypass surgery. Also, the content of the thrombus in the two long bypass grafts was abundant and thrombolysis seemed difficult. Therefore, EVT was tried for a native occluded CIA to the SFA.

First EVT: Initial intravenous injection was 5000 U of heparin, and >250 seconds of activated clotting time (ACT) was kept every 1 hour.

**Fig. 2:**
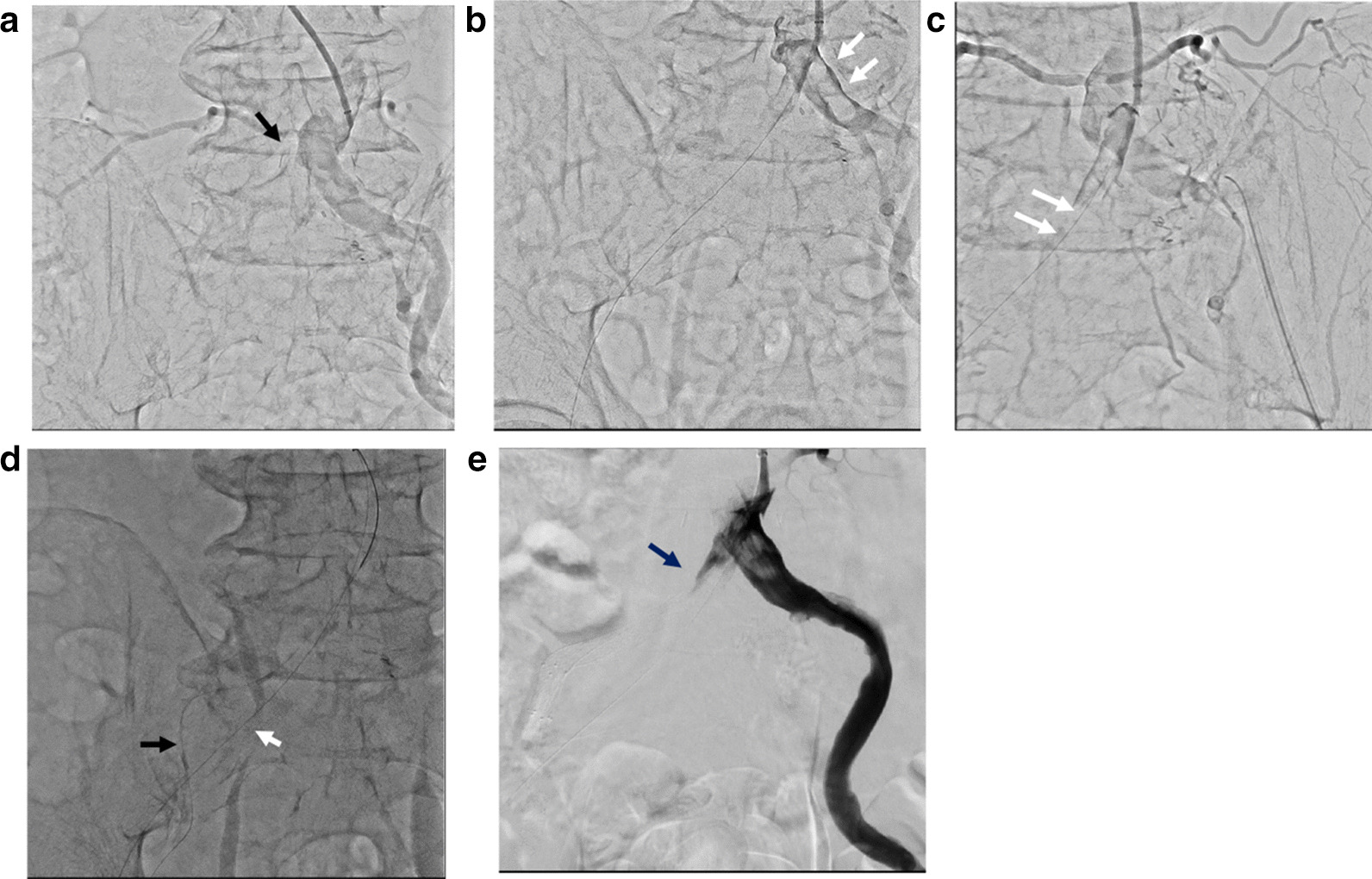
First endovascular therapy at the second hospitalization. **a** The native common iliac artery (CIA) and the right aortofemoral bypass were occluded (black arrow). **b** After ballooning the failed prosthetic aortofemoral bypass graft, the thrombus moved to the left CIA (white arrow). **c** The guidewire was found in the aortofemoral graft with a large amount of thrombus (white arrow). **d** The soft guidewire (black allow) in the native iliac artery was externalized by intravascular ultrasound guidance. A guidewire in the aortofemoral bypass graft (white arrow) was a good landmark. **e** Implanting two stents from the CIA to the EIA did not result in sufficient flow (black arrow)

Antegrade approach (1): A 6-Fr 90-cm Destination sheath (Terumo Medical Corporation, Tokyo, Japan) was inserted from the left brachial artery to the abdominal aorta. A 0.014-inch guidewire (Herbert, Asahi Intecc Co., Aichi, Japan) was unable to penetrate the occluded CIA (Fig. 2a)

Retrograde approach (1): A 4-Fr 30-cm sheath was also inserted from the right popliteal artery (PA). A 0.014-inch guidewire (Gladius, Asahi Intecc Co.) with a microcatheter was passed from the PA to the abdominal aorta, where balloon angioplasty was performed from retrograde with a semicompliant balloon (Coyote™, 3.0 × 220 mm, Boston Scientific, Tokyo, Japan). The thrombus unexpectedly transferred to the left CIA after balloon dilation, nearly occluding it (Fig. 2b). An angiography and intravascular ultrasound (IVUS) confirmed that the guidewire was through the native SFA to the thrombus-rich aortofemoral graft (Fig. 2c). It appeared that the thrombus had moved from the aortofemoral graft to the native left CIA as the balloon was inserted and removed. An 8-Fr 10-cm sheath was then inserted into the left common femoral artery (CFA), and thrombectomy was performed using a Dio catheter (Nipro Co., Osaka, Japan). The thrombus was then almost completely removed from the left CIA.

Retrograde approach (2): Inserting the sheath directly from the proximal SFA to the EIA with body surface ultrasonography guidance rather than passing the guidewire from the obstructed distal SFA to the EIA was thought to be easier. Therefore, the inside of the SFA CTO site was punctured and a 6-Fr 10-cm sheath to the right EIA was inserted. A 0.014-inch Gladius guidewire with microcatheter was inserted from a retrograde direction, but proceeded to the subintima.

Antegrade approach (2): A microcatheter with a 0.014 hard guidewire (Chevalier^®^ tapered 30, Cardinal Health, Tokyo, Japan) successfully penetrated the right CIA. The guidewire in the aortofemoral bypass was a good landmark to penetrate the CIA (Fig. 2d). The antegrade guidewire was able to be joined to the retrograde wire at the same occluded true lumen at the EIA by IVUS guidance. Additionally, the soft wire (Chevalier Universal 300, Cardinal Health Japan) was externalized (Fig. 2d).

Retrograde approach (3): Two self-expandable nitinol stents (S.M.A.R.T. Control, 8 × 80 and 7 × 60 mm, Cardinal Health Japan) were retrogradely implanted from the CIA to the EIA.

Antegrade approach (3): Antegrade crossing of the guidewire from the EIA to the SFA was then attempted. However, it was not successful.

Retrograde approach (4): The repeated balloon angioplasty in the SFA with a semicompliant balloon (Sterling™, 5.0 × 150 mm, Boston Scientific) was finalized using the guidewire which crossed from the SFA to the aortofemoral bypass. However, efficient flow could not be obtained from the native CIA to the SFA (Fig. 2e).

Heparin and PGE1 infusion were continued after EVT. However, the cold sensation in the patient’s lower limbs did not resolve. Thus, we performed a second EVT 2 days later.

Second EVT: Heparin (5000 U) was intravenously injected, and kept > 250 seconds of ACT every 1 hour.

**Fig. 3:**
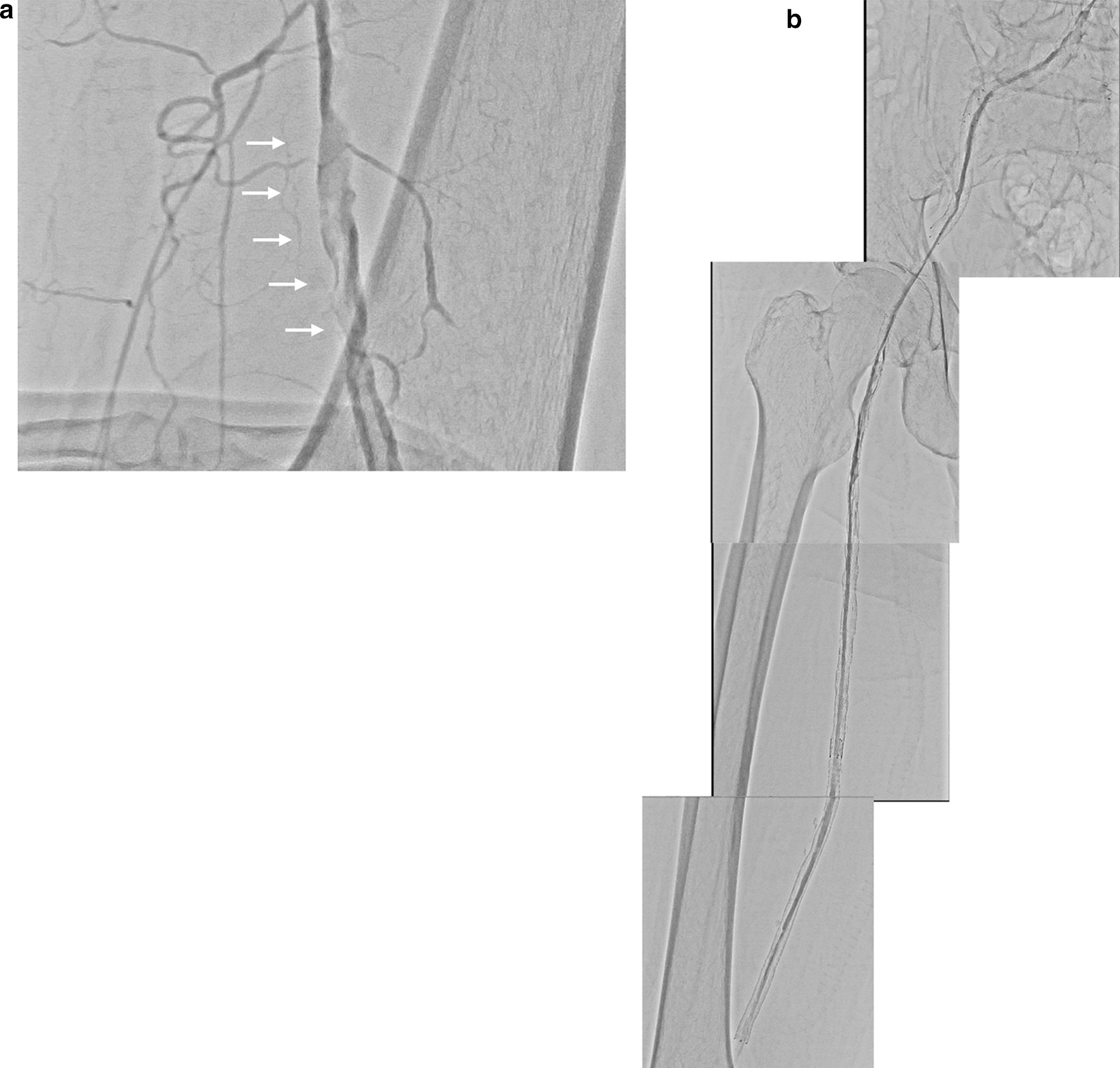
Second endovascular therapy. **a** The native distal superficial femoral artery had a massive thrombus (white arrows). **b** Two self-expandable nitinol stents were implanted in the right SFA. However, the thrombus remained and the iliofemoral artery flow was insufficient

Retrograde approach: When a 6-Fr 30-cm sheath was inserted in the right PA, a massive thrombus was found in the distal SFA (Fig. 3a). First, the thrombus was aspirated with an aspiration catheter (8-Fr Thrombuster II, Kaneka Co., Osaka, Japan). A microcatheter with a 0.014-inch Gladius guidewire was then easily able to turn from the SFA to the aortofemoral graft. Using IVUS guidance, a double lumen catheter with a hard guidewire (Chevalier^®^ tapered 30, Cardinal Health) was crossed to the previous EIA stenting site by parallel wiring. After angioplasty was performed with a semicompliant balloon (Sterling™, 6.0 × 150 mm, Boston Scientific) from the CIA to the SFA, IVUS showed a large amount of thrombus remaining in the SFA. Repeated aspiration using a thrombectomy catheter, long inflation of a semicompliant balloon, and inflation of a scoring balloon (NSE PTA, 7.0 × 40 mm, Nipro Co., Osaka, Japan) did not result in recanalization. Therefore, two more stents (LifeStent Solo, 7.0 × 200 and 6.0 × 150 mm, Medicon Inc., Osaka, Japan) were implanted in the right SFA, avoiding the CFA. Thrombectomy was then repeated. Eventually, a small amount of in-line flow was obtained from the CIA to the SFA. However, it was insufficient due to residual thrombus in the stents (Fig. 3b).

**Fig. 4:**
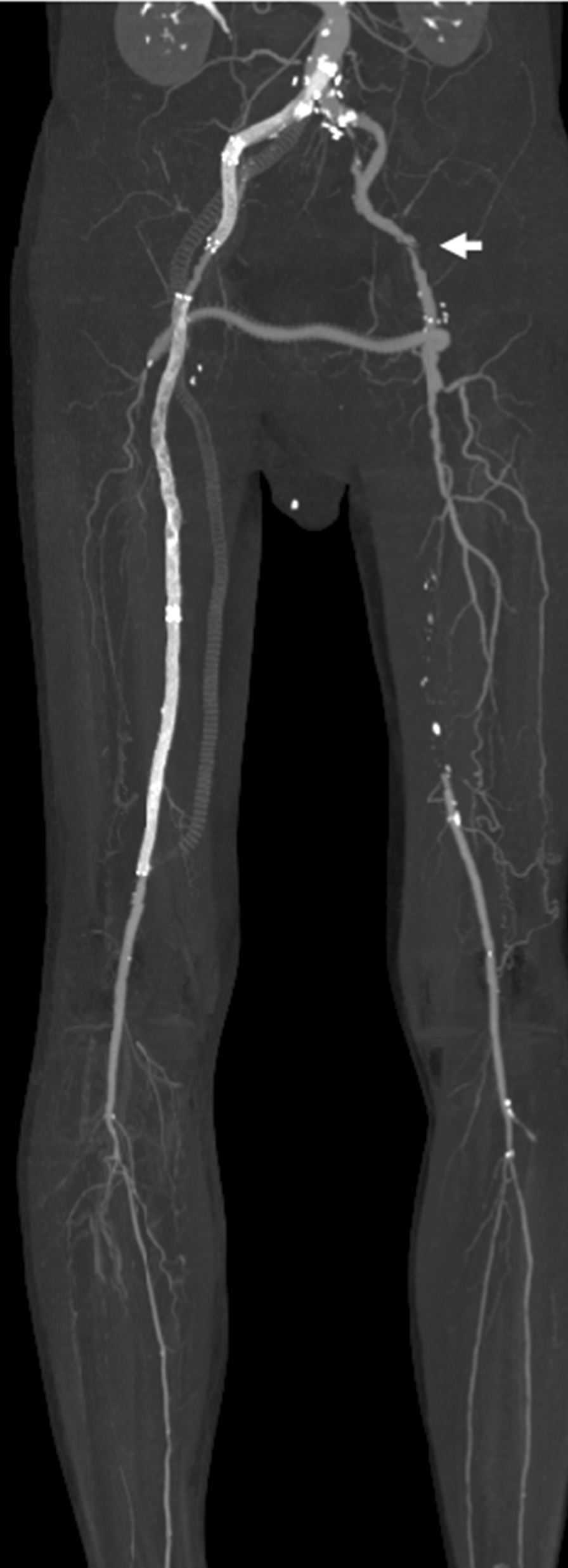
Multislice computed tomography (MSCT) after a rescue left common femoral-right deep femoral artery bypass. Good blood flow but remaining thrombus in the implanted stents and patency of femorofemoral bypass were found. The left external iliac artery had moderate stenosis (white arrow)

Heparin and PGE1 infusion were continued after EVT. The patient’s resting pain improved, but the cold sensation did not. Therefore, a rescue left CFA-right deep femoral artery (DFA) bypass using PTFE (6 mm, Gore Propaten) was performed 4 days after the second EVT. The cold sensation in the lower limbs gradually improved after surgery. Moreover, MSCT confirmed the patency of the femorofemoral bypass graft to the left DFA and good blood flow with a small amount of residual thrombus in the implanted iliofemoral stents 7 days after surgery (Fig. 4).

The patient showed no obvious coagulation abnormality. The levels of protein C antigen (81%), protein S antigen (90%), and antithrombin III (AT-III) (81%) were in the normal range. Platelet factor 4 (PF-4)-heparin complex antibody (< 0.6 U/ml) and antiphospholipid antibody (1.0 ratio) were negative. The patient received dual antiplatelet therapy of aspirin (100 mg per day) + cilostazol (200 mg per day), telmisartan (40 mg per day), amlodipine (5 mg per day), and rosuvastatin (5 mg per day) after femorofemoral bypass.

**Fig. 5:**
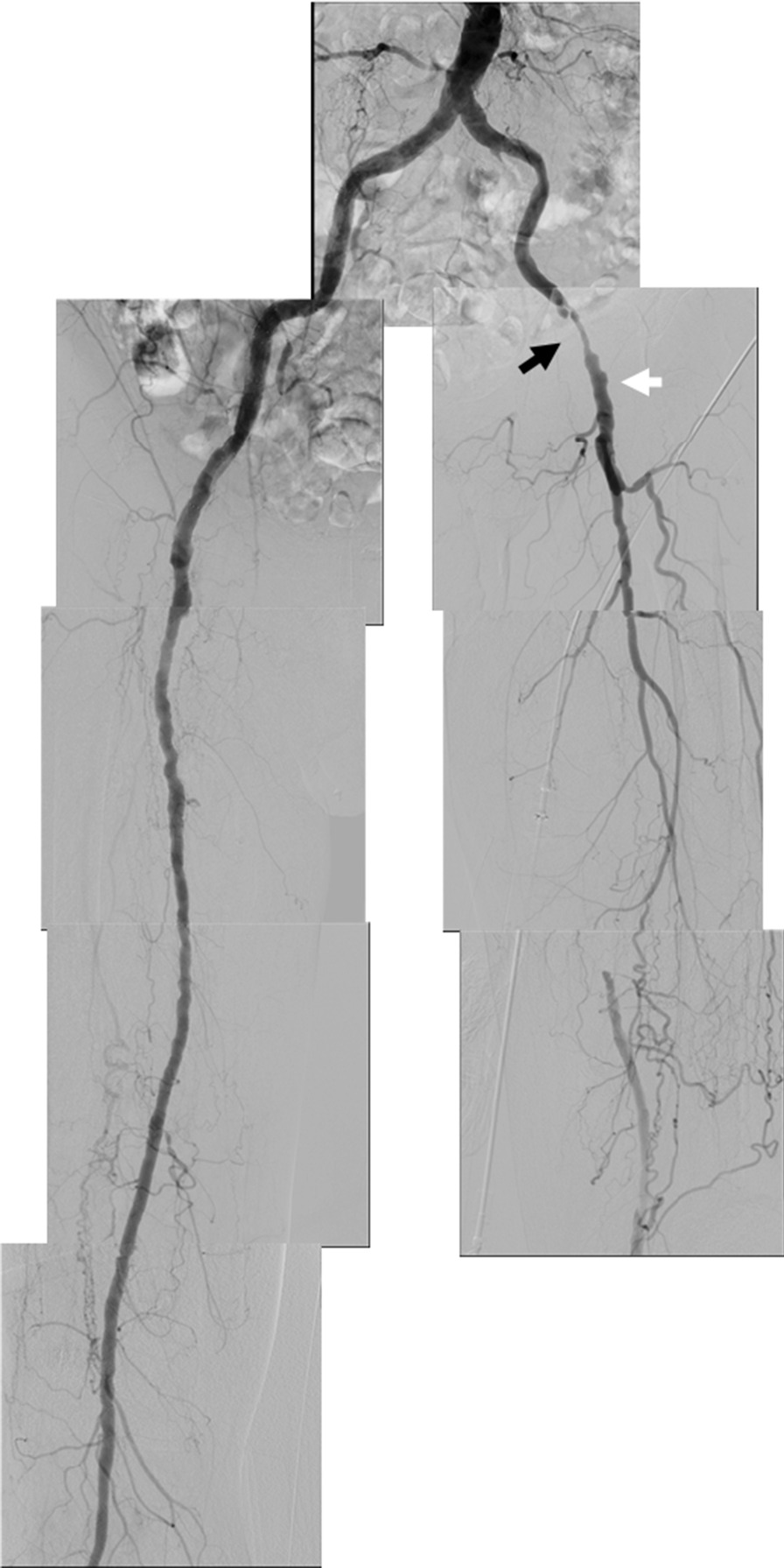
Lower extremity angiography 5 months after a rescue femorofemoral bypass. Good flow was found in the implanted stents from the right CIA to SFA. However, the femorofemoral bypass graft was occluded (white arrow) due to progression of the left distal EIA stenosis (black arrow)

He developed worsening of intermittent claudication in the left leg 5 months later. Angiography showed a good flow in the implanted stents in the right CIA to the SFA (Fig. 5). However, the femorofemoral bypass graft was unfortunately occluded because of the progression of the left distal EIA stenosis (Fig. 5). Balloon angioplasty was performed in the left EIA to the CFA. An IVUS also confirmed the disappearance of the thrombus in the implanted right iliac stents. The patency of the implanted right iliofemoral stents was confirmed at 1 year by ultrasonography. However, three drug-eluting stents (Eluvia™ 6.0 × 120, 6.0 × 120, 7 × 80 mm, Boston Scientific) were implanted at that time in the occluded left SFA because of worsening of intermittent claudication again in the left leg.

## Discussion

We experienced a case of ALI due to failed aortofemoral and femoropopliteal bypass grafts. Stent implantation in the long CTO from the CIA to the SFA did not result in efficient flow due to thrombus movement from the aortofemoral graft. However, a rescue femorofemoral bypass dissolved the thrombus in the implanted iliofemoral stents. To the best of our knowledge, there are no studies reporting that the residual thrombus within implanted stents can be dissolved by increasing the collateral circulation. This phenomenon may contribute to future treatment strategies for thrombus dissolution without the use of thrombolytic drugs.

Failed aortofemoral and femoropopliteal bypass grafts in the lower extremity artery result in ALI. Fogarty balloon thromboembolectomy has remained the standard treatment for ALI caused by embolic occlusion. However, residual thrombus may limit the clinical success rate. Surgical revascularization followed by endovascular techniques such as stent implantation, CDT, and thrombectomy by aspiration catheter (so-called hybrid procedures) have improved limb salvage and reintervention rates [7]. EVT has been found to have better short-term outcomes, such as avoiding transfusion, major amputation, and short-term reintervention, compared with open surgery and hybrid revascularization [8].

The patient had undergone anatomical bypass 1 year before, with intestinal hernia complication after surgery. Thus, his surgical risk was surely adequate to undergo a second open procedure. Thrombectomy in the occluded aortofemoral bypass seemed to be difficult because of the suspected massive thrombus in the aortofemoral graft. CDT for occluded grafts was not chosen to avoid the risk of bleeding, because the patient had a bladder carcinoma. Therefore, EVT was first tried for the occluded native iliofemoral artery. Unfortunately, however, the retrograde guidewire passed through the prosthetic aortofemoral graft, resulting in thrombus movement and inefficient in-line flow after stent implantation in the CIA to the SFA. Thrombectomy from the CIA to the SFA was performed to the greatest extent possible using a thrombectomy catheter. However, it was insufficient. More aggressive thrombus removal may be achieved if mechanical thrombectomy devices (such as AngioJet™, Jetstream™, and Penumbra™) could have been used.

By performing a rescue femorofemoral bypass to the DFA, the stent-implanted iliofemoral artery flow gradually improved. This seems to have spontaneously dissolved the thrombus in the stents due to the increased blood flow to the periphery by the femorofemoral bypass. When collateral circulation to the periphery of the occluded blood vessel increases and the thrombus amount is not so large, the thrombus in the occluding blood vessel may be dissolved. This may help future treatment strategies to dissolve a thrombus without the need for CDT.

The rescue femorofemoral bypass was unfortunately occluded instead of the thrombus in the stents in the iliofemoral artery being dissolved. The stenosis in the left EIA remained, which was insufficient for long-term patency as an inflow of femorofemoral bypass. The left EIA intervention should have been made earlier. Alternatively, axillary-femoral bypass or femorofemoral bypass, which is an extra-anatomical surgical approach, should have been performed first instead of complex EVT. Consequently, the lateral axillary-DFA bypass rescued ALI due to the failed bilateral axillary-femoral bypass grafts [9].

So far, the patient has no symptoms such as intermittent claudication in the right leg. However, his IIA and DFA, which are important collateral sources for the lower limbs, are occluded. If the implanted stents in the iliofemoral artery are occluded, ALI could again occur. Careful follow-up is needed to determine whether the ongoing use of double antiplatelet therapy (aspirin and cilostazol) and statin could maintain the long-term patency in the implanted stents.

## Conclusion

Treating this long CTO from the CIA to the SFA was difficult. The unexpected thrombus movement caused by balloon dilation in the failed graft further increased the difficulty. A femorofemoral bypass was important to rescue ALI in the failed aortofemoral and femoropopliteal grafts, and the increased peripheral collateral blood flow through the DFA dissolved the residual thrombus in the implanted stents of the iliofemoral artery.

## Data Availability

Not applicable.

## Abbreviations

ALI: Acute limb ischemia
CIA: Common iliac artery
IIA: Internal iliac artery
CFA: Common femoral artery
SFA: Superficial femoral artery
DFA: Deep femoral artery
PA: Popliteal artery
MSCT: Multislice computed tomography

## References

[1] Rutherford RB, Baker JD, Ernst C, Johnston KW, Porter JM, Ahn S, et al. Recommended standards for reports dealing with lower extremity ischemia: revised version. J Vasc Surg. 1997;26:517–38. 10.1016/s0741-5214(97)70045-4 9308598

[2] Nolan KD, Benjamin ME, Murphy TJ, Pearce WH, McCarthy WJ, Yao JS, et al. Femorofemoral bypass for aortofemoral graft limb occlusion: a ten-year experience. J Vasc Surg. 1994;19:851–6. 10.1016/s0741-5214(94)70010-9 8170039

[3] Veenstra EB, van der Laan MJ, Zeebregts CJ, de Heide EJ, Kater M, Bokkers RPH. A systematic review and meta-analysis of endovascular and surgical revascularization techniques in acute limb ischemia. J Vasc Surg. 2020;71:654–68. 10.1016/j.jvs.2019.05.031 31353270

[4] Higashitani M, Anzai H, Mizuno A, Utsunomiya M, Umemoto T, Yamanaka T, et al. One-year limb outcome and mortality in patients undergoing revascularization therapy for acute limb ischemia: short-term results of the Edo registry. Cardiovasc Interv Ther. 2020. 10.1007/s12928-020-00662-6. 32212046

[5] Ouriel K, Veith FJ, Sasahara AA. A comparison of recombinant urokinase with vascular surgery as initial treatment for acute arterial occlusion of the legs. Thrombolysis or Peripheral Arterial Surgery (TOPAS) Investigators. N Engl J Med. 1998;338:1105–11. 10.1056/NEJM199804163381603 9545358

[6] Theodoridis PG, Davos CH, Dodos I, Iatrou N, Potouridis A, Pappas GM, et al. Thrombolysis in acute lower limb ischemia: review of the current literature. Ann Vasc Surg. 2018;52:255–62. 10.1016/j.avsg.2018.02.030 29772326

[7] de Donato G, Setacci F, Sirignano P, Galzerano G, Massaroni R, Setacci C. The combination of surgical embolectomy and endovascular techniques may improve outcomes of patients with acute lower limb ischemia. J Vasc Surg. 2014;59:729–36. 10.1016/j.jvs.2013.09.016 24342067

[8] Davis FM, Albright J, Gallagher KA, Gurm HS, Koenig GC, Schreiber TP, et al. Early outcomes following endovascular, open surgical, and hybrid revascularization for lower extremity acute limb ischemia. Ann Vasc Surg. 2018;51:106–12. 10.1016/j.avsg.2017.12.025 29518503

[9] She K, Zhang X, Yin J, Cheng G, Chen X, Zhang Y. Case report: lateral axillary-profunda femoris artery bypass for acute lower limb ischemia due to thrombosis after bilateral axillofemoral bypass. J Cardiothorac Surg. 2020;15:206. 10.1186/s13019-020-01232-w. PMC7394680 32736648

